# Correlation between serum albumin and serum zinc in malignant lymphoma

**DOI:** 10.20407/fmj.2021-006

**Published:** 2021-08-20

**Authors:** Misaki Morisaku, Kaori Ito, Anna Ogiso, Misa Imai, Yoshiko Hiraoka, Miho Zennami, Masahiro Tsuge, Maiko Mori, Seira Toyosato, Hidezo Matsuda, Yosuke Ando, Masutaka Tokuda, Akihiro Tomita, Shigeki Yamada

**Affiliations:** 1 Department of Clinical Pharmacy, Fujita Health University, School of Medicine, Toyoake, Aichi Japan; 2 Department of Pharmacy, Fujita Health University Hospital, Toyoake, Aichi, Japan; 3 Department of Hematology, Fujita Health University, School of Medicine, Toyoake, Aichi, Japan

**Keywords:** Zn deficiency, Malignant lymphoma, Serum albumin level

## Abstract

**Objectives:**

Zinc (Zn) is a cofactor for more than 200 enzymes within the human body. Zn deficiency can result in cell-mediated immune dysfunction. Furthermore, serum Zn levels have been reported to be associated with nutritional status, but this association has not been clarified in malignant lymphoma. This study aimed to examine the deficiency of serum Zn levels and clarify the factors that are correlated with serum Zn in malignant lymphoma.

**Methods:**

Initial malignant lymphoma was diagnosed in patients at Fujita Health University Hospital between April 2011 and March 2019. Based on the serum Zn levels, the study population was divided into “deficient” and “low or normal”. For the serum Zn levels of patients undergoing pre-chemotherapy, laboratory parameters and nutritional factors were included. We compared these factors between the abovementioned two groups, and the serum Zn levels with its correlation factors were investigated.

**Results:**

A total of 77 patients (Deficient group, n=20 and Low or Normal group, n=57) were enrolled. Histology, hemoglobin, serum albumin levels, Glasgow Prognostic Score (GPS), neutrophile-lymphocyte ratio (NLR), prognostic nutrition index (PNI) and Controlling Nutritional Status (CONUT) were significantly different between the two groups. Of these parameters, only serum albumin level was significantly associated with serum Zn level (p=0.0024; estimated regression coefficient, 9.51; adjusted coefficient of determination, 0.28).

**Conclusions:**

Poor nutritional status at the initial diagnosis may have affected Zn deficiency in initial malignant lymphoma.

## Introduction

Zinc (Zn) is an essential trace element required for various biological processes in the human body. Zn has functional roles in enzyme activation and immune function.^1–4^ The five distinct phases of an immune response include: recognition, activation, proliferation, effector function, and memory.^5^ Zn deficiency is associated with protein-energy malnutrition in the elderly.^6^ Zn is involved in various diseases and plays different roles in maintaining a healthy status of humans. Some of the examples are as follows: Zn metabolism is disrupted in liver cirrhosis^7^; serum Zn concentration is involved in chronic liver diseases, as reported by Nishikawa *et al.*^8^; a decrease in serum Zn levels is used for prognosis prediction in acute myocardial infarction, as reported by Singh *et al.*^9^; Zn administration improves renal anemia in erythropoietin-refractory renal anemia patients, as reported by Fukushima *et al.*^10^; and Zn deficiency anemia in blood diseases.^11^

In malignant tumors, tumor-induced expenditure and a decrease in the nutritional state may reduce serum Zn levels, but the details of this mechanism remain unknown. Active oxygen can act as a direct or an indirect cause of Zn reduction in lifestyle diseases, aging, and carcinogenic processes. Active oxygen is involved in the onset or exacerbation of 90% of diseases, and Zn is a strong, active oxygen-inhibiting factor. Metals such as Cu, Zn, Mn, Fe, and Se are bound to the active center of superoxide dismutase, an active oxygen-scavenging enzyme. Previous reports have revealed an association between Zn deficiency and low T-lymphocyte count in head and neck cancer patients.^12,13^ However, in malignant lymphoma, it is unknown whether decreased Zn is specific to this disease.

In a previous study, we reported Zn deficiency in patients with hematological malignancies.^14^ Furthermore, Zn deficiency reported also cause dermatitis, diarrhea, depression, impaired taste and decreased appetite in elderly.^15^ Zn deficiency can adversely affect malignant lymphoma. However, the relationship between serum Zn levels and peripheral blood factors and nutritional factors has not been established in lymphoma patients. Thus, in this survey we aimed to investigate the relationship between those factors and serum Zn levels as well as the factors affecting for Zn level in lymphoma patients. Understanding these relationships, and whether Zn supplementation can result in the recovery of the associated factors is of importance in the context of lymphoma patients.

## Methods

### Subjects

Patients with an initial diagnosis of malignant lymphoma, whose Zn serum levels had been measured prior to undergoing chemotherapy, were included in this study. Patients included were those admitted to the Division of Haematology at Fujita Health University Hospital between April 2011 and March 2019. Patients who had taken any Zn containing medication were excluded from the study, as were patients who had undergone gastric and/or ileocecal resection.

### Investigations

Date on patient characteristics included in the analysis were: age, sex, performance status (PS), clinical stage (CS), histology, height (HT), body weight (BW), body surface area (BSA), body mass index (BMI) and laboratory parameters (white blood cell, hemoglobin level, total lymphocyte counts (TLC), CD3^+^, CD4^+^, CD8^+^ and CD20^+^ lymphocyte counts). As nutritional factors, we investigated serum albumin levels (Alb), Glasgow Prognostic Score (GPS), neutrophil-lymphocyte ratio (NLR), platelet-lymphocyte ratio (PLR), prognostic nutrition index (PNI) and controlling nutritional status (CONUT), were obtained from electronic medical records. GPS was calculated by combining C-reactive protein and Alb levels^16^; PNI was calculated using the formula PNI=10×Alb+0.005×TLC^17^; and CONUT was calculated using Alb, TLC, and total cholesterol levels.^18^

According to facility reference values and previous reports, we defined two groups of Zn deficiency. The serum Zn levels higher than or equal to 80 μg/dL were defined as “normal,” levels between 60 and 79 μg/dL were defined as “low,” and levels lower than 60 μg/dL were defined as “deficient”. We defined the two groups as “deficient” and “low or normal” groups.^19,20^ We investigated the comparison of peripheral blood and nutritional marker between the two groups. The correlation between serum Zn level and age, BSA, BMI, peripheral blood factors, and nutritional factors was also examined.

### Determination of serum Zn levels and lymphocyte subsets

Serum Zn levels were measured by BM6010 analysis (JEOL Ltd., Tokyo, Japan) using 2- (5-bromo-2-pyridylazo)-5-(N-propyl-N-sulfopropylamino) phenol; a chelate compound of Zn. CD4^+^, CD8^+^ and CD20^+^ lymphocyte counts were established using a Cytomics FC500 (Beckman Coulter Inc., Calif., USA). For measurement of lymphocyte counts, we used an FITC-labelled CD4 murine monoclonal IgG antibody fraction, a PE-labelled CD8 murine monoclonal IgG antibody fraction, and a PE-labelled CD20 murine monoclonal IgG antibody fraction (Beckman Coulter).

### Statistical analysis

Normally distributed variables were expressed as means±standard deviations, whereas non-normally distributed variables were expressed as medians and interquartile ranges. Chi-square or Fisher’s exact tests and Mann-Whitney U-tests were used to analyse the relationship between Zn levels and categorical variables and continuous variables, respectively. Spearman’s correlation coefficient was used to evaluate correlations among continuous variables. Zn levels related other factors considered with a correlation coefficient r_s_>0.2. Linear regression was used to investigate the multivariate associations with serum Zn levels. Explanatory variables were selected from significant factors (*p*<0.05) in the comparison between the two groups, in addition to age, which was an important factor in the previous studies.^21^ The R Statistical Package (The R Foundation for Statistical Computing, Vienna, Austria version 3.5.2) was used for all statistical analyses, and differences with p<0.05 were considered significant.

### Ethics

The present retrospective study was approved by the Fujita Health University School of Medicine Epidemiological and Clinical Research Ethics Committee. All procedures performed in this study involving human participant were in accordance with the ethical standards of the institutional and/or national research committee and with the Declaration of Helsinki and its later amendments or comparable ethical standards. There was a limitation in gathering information because this study involved a retrospective investigation. We confirmed the tolerance level in the extent necessary in daily and gathered information on the condition, which was constant.

## Results

### Patients

A total of 77 patients were identified and classified as Zn deficient (n=20), low Zn levels (n=45), and normal Zn levels (n=12). These baseline characteristics of the 77 patients included in our analysis are listed Table 1. There were no statistically significant differences in age, sex, PS, CS, HT, BW, BSA, and BMI between the two groups. The histology was significantly different between the two groups. Median Zn levels were 67 (61–75) μg/dL for patients aged below 75 years (n=50) and 63 (58–73) μg/dL for patients aged above 75 years (n=27), with no significant differences between the two groups. There were no significant differences in serum Zn level among patients with diffuse large B-cell lymphoma, and follicular lymphoma.

### Comparison of peripheral blood and nutritional marker in two groups

Hemoglobin, serum albumin levels, GPS, NLR, PNI and CONUT were significantly different between the two groups (Table 2). There were no significant differences in white blood cell counts, TLC, CD3^+^ lymphocyte, CD4^+^ lymphocyte, CD8^+^ lymphocyte, CD20^+^ lymphocyte, PLR between the two groups.

### Association between serum Zn level and peripheral blood factors

**Figure 1:**
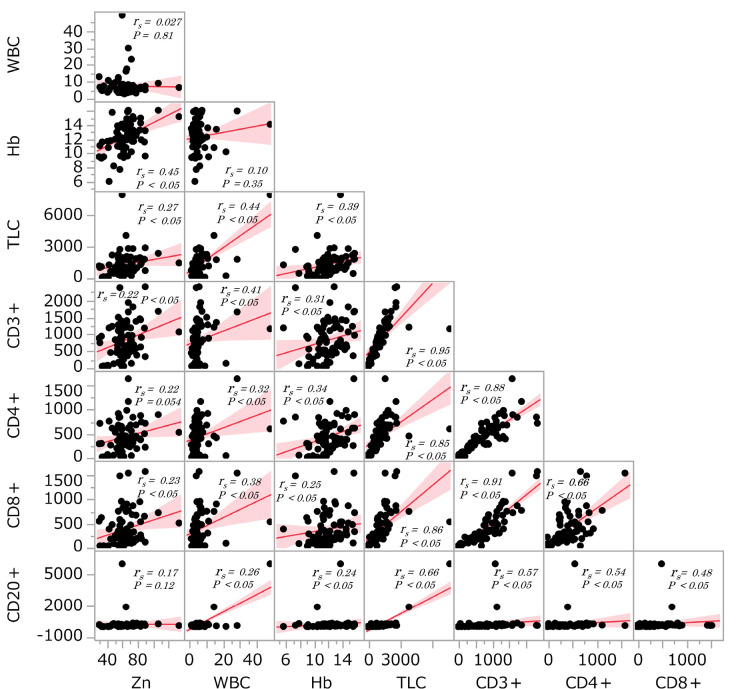
Spearman correlation analysis of serum Zn level and peripheral blood factors. Correlations between serum Zn levels and peripheral blood factors including white blood cell, hemoglobin, total lymphocyte count (TLC), CD3^+^, CD4^+^, CD8^+^and CD20^+^ lymphocyte counts.

The correlations between serum Zn level and other peripheral factors such as white blood cell count, hemoglobin, TLC, and CD3^+^, CD4^+^, CD8^+^ and CD20^+^ lymphocyte counts were analysed (Figure 1). When the peripheral factors were assessed as continuous variables, there were weak but significant positive associations between serum Zn levels and total lymphocyte count (r_s_=0.27, p<0.05), haemoglobin (r_s_=0.45, p<0.05), CD3^+^ lymphocyte count (r_s_=0.22, p<0.05), and CD8^+^ lymphocyte count (r_s_=0.23, p<0.05). CD4^+^ lymphocyte count was weakly and not significantly associated with serum Zn levels (r_s_=0.22, p=0.054). White blood cell count and CD20^+^ lymphocyte count had no correlation with Zn levels (r_s_=0.027, p=0.814, r_s_=0.17, p=0.12).

### Association between serum Zn level and nutritional factors

**Figure 2:**
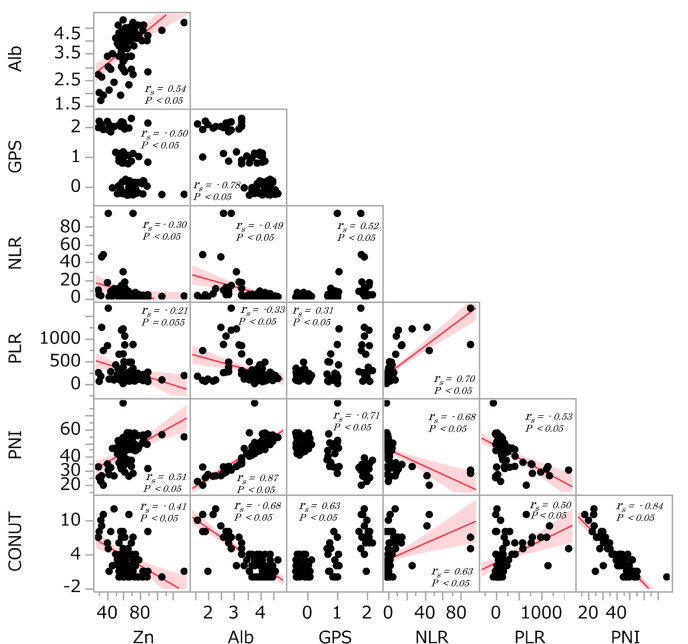
Spearman correlation analysis of serum Zn level and peripheral nutritional factors. Correlations between serum Zn levels and peripheral nutritional factors including albumin (Alb), Glasgow Prognostic Score (GPS), neutrophile-lymphocyte ratio (NLR), platelet-lymphocyte ratio (PLR), prognostic nutrition index (PNI) and Controlling Nutritional Status (CONUT).

The correlations between serum Zn level and other nutritional factors such as serum Alb, GPS, NLR, PLR, PNI and CONUT were analysed (Figure 2). When the nutritional factors were assessed as continuous variables, serum albumin levels were positive associated with serum Zn levels (r_s_=0.54, p<0.05) and PNI (r_s_=0.51, p<0.05). There were significant negative associations between GPS (r_s_=–0.50, p<0.05), NLR (r_s_=–0.30, p<0.05) and CONUT (r_s_=–0.41, p<0.05) and Zn levels.

### Affecting factors

The factors appearing to affect serum Zn level were further, examined using multiple linear regression analysis. Serum Zn level was used as the response variable and age, NLR and hemoglobin level as explanatory variables. GPS, PNI, and CONUT, which used Alb in their calculations, were excluded from the explanatory variables because of multicollinearity with Alb. Only serum albumin level was significantly associated with serum Zn level (p=0.0024; estimated regression coefficient, 9.51; adjusted coefficient of determination, 0.28) (Table 3).

## Discussion

In the present study, 84.3% of patients with malignant lymphoma had low or deficient serum Zn levels, and one quarter of these patients were in a state of Zn deficiency. These results corresponded with those of our previous study.^14^ In addition, serum Zn levels were positively correlated with serum albumin levels, total lymphocyte count, CD3^+^ and CD8^+^ lymphocyte counts and PNI. Multiple linear regression analysis revealed serum albumin level to be a significant affecting factor.

One reason for the apparent relationship between albumin and Zn levels could be due to a decrease in albumin resulting in a decrease in protein-bound Zn levels. In the blood, albumin and 60%–70% of absorbed Zn exists as loosely bound elements. A lack of Zn intake or increased urinary excretion of Zn induced by hypoalbuminemia may also contribute to this relationship. Reserve proteins, such as ferritin in iron, cannot bind to Zn. With regard to Zn absorption, Kambe et al. reported that Zip4 and Zip5, which are Zn transporters in small intestinal epithelial cells, are important for Zn balance and are involved in Zn ion homeostasis. Insufficient Zn levels increase Zip4 expression and prevent degradation of Zip4 expression.^22^ In a state of undernutrition, this absorption mechanism does not function normally, and Zn homeostasis maintenance in the body may fail.

In the present study, median serum Zn levels of patients presenting with lymphoma for the first time were 65 (59–75) μg/dL. The median age was 68 (59–77), with 27 patients (35.0%) aged above 75 years. Compared with the mean value of 87.5±11.2 (min 65, max 110) μg/dL, which is the reference value of serum Zn levels in healthy human body, Zn levels were 20 μg/dL lower in these patients with lymphoma. The NHANES II study conducted in the United States from 1976 to 1980 reported Zn levels in 14,770 people from the general population.^23^ NHANES II revealed that the Zn levels (mean±standard deviation, μg/dL) were 89.1±0.63 for males aged 45–64 years, 85.6±0.79 for males aged 64–74 years, and 84.4±0.53 for females aged 45–64 years, 83.5±0.53 for females aged 65–74 years. The results of our study clearly show that serum Zn levels are lower than those in the survey in the United States, even though conditions, such as specimen collection methods, measurement precision of serum Zn levels, racial differences or differences in diet and other lifestyle parameters, did not differ between our study and the survey. In addition, as serum Zn levels decrease with age, it is necessary for the elderly to be aware of the possibility of Zn deficiency. In Japan, Kurasawa et al. investigated the serum Zn concentration of 1,431 Japanese individuals and reported that in 20% of all adults, Zn levels were below 65 μg/dL, at the lower limit of the reference value, and many patients showed Zn deficiency as they aged.^21^ The elderly population is prone to Zn deficiency, and Zn supplementation of 15 mg per day, the adult requirement, or more, is necessary. It has also been reported that Zn deficiency is more frequent particularly in those over 75 years-old. Patients above the age of 75 constituted 35.0% of all subjects in this study. However, albumin level was shown to be an independent factor affecting serum Zn level by multiple linear regression analysis; thus, it is possible to exclude the effects of age.

For all cases in this study, measurement of serum Zn levels was performed at the regular blood collection laboratory every morning during clinical practice. Zn levels in the blood comprise only about 1% of whole body Zn content, and physical activities and stress can cause an internal shift to the liver and other tissues. Serum Zn concentration has been reported to be at its maximum at 8 a.m. and at its minimum at 3 p.m.^24^ In the Japanese survey, for all adult age groups, Zn levels were higher in cases where blood collection occurred in the morning (532 cases) than in the afternoon (551 cases), with Zn levels (mean±standard deviation) at 75.0±12.3 μg/dL and 65.2±9.7 μg/dL, respectively. Individuals who underwent morning blood collection, on average had Zn levels around 10 μg/dL higher than those of who underwent blood collection in the afternoon, suggesting the existence of circadian variation in the population.^23^

Zn levels in red blood cells are 10-fold higher compared with those in serum, and Zn in white blood cells are 10-fold higher compared with those of red blood cells.^25^ Thus, when measuring serum Zn, it is necessary to perform serum separation rapidly after blood collection to prevent the elution of Zn in blood cells. Although some advocate using Zn concentration in white blood cells in the diagnosis of Zn deficiency, it is common to use serum Zn as it is clinically easy to measure. In this study, blood collection was performed before 8 a.m., when serum Zn level is expected to be closer to the maximum value.

Takeda et al. have shown that many enzymes involved in the degradation of extracellular adenosine triphosphates (ATPs) are Zn-requiring enzymes. As such, Zn insufficiency causes the accumulation of ATPs outside of cells, as well as a decrease in adenosine, a degradation product of ATP, causing various symptoms of Zn deficiency.^26^ Lower serum Zn levels are associated with higher possibility of developing Zn deficiency, although high values within the “reference value” may lead to Zn deficiency. Therefore, it is important to compare serum Zn levels with clinical symptoms and perform an integrated evaluation. We believe that such an evaluation should be applied to patients with malignant lymphoma.

There are several limitations to the present study. First, this study was observational study and not a randomized controlled trial. Second, we did not measure other trace elements such as copper and iron. However, to the best of our knowledge, this study is the first to report that Alb correlates with serum Zn in malignant lymphoma. Poor nutritional status at initial diagnosis may have affected serum zinc levels in lymphoma patients.

## Figures and Tables

**Table1:** Patients’ characteristics

	All (n=77)	Deficient (n=20)	Low or Normal (n=57)	P-value
Age median (years)	68.0 (59.0–77.0)	73.0 (58.7–77.5)	67.0 (59.0–76.0)	0.40
Sex Male/Female	40/37	9/11	31/26	0.60
PS (0–2/3, 4)	58/19	11/9	47/10	0.11
CS (1/2/3/4)	11/15/8/43	2/5/2/11	9/10/6/32	0.91
Histology				
HL	1	0	1	
NHL	76	20	56	
B cell	71	17	54	
DLBCL/FL/others	47/10/14	17/0/0	30/10/14	0.001
T cell	4	2	2	
NK cell	1	1	0	
HT (cm)	159 (151–166)	156 (150–165)	160 (151–166)	0.38
BW (kg)	51.2 (45.7–60.1)	53.1 (45.2–56.3)	51.0 (45.7–61.7)	0.54
BSA (m^2^)	1.5 (1.3–1.6)	1.5 (1.3–1.6)	1.5 (1.3–1.6)	0.61
BMI (kg/m^2^)	20.9 (18.9–23.1)	20.3 (19.2–22.9)	21.0 (18.9–23.1)	0.41
Serum Zn level (μg/dL)	65.0 (59.0–75.0)	51.0 (42.7–57.2)	70.0 (63.0–77.0)	<0.001

Variables that did conform to a normal distribution are expressed as the median (interquartile range). PS, Performance Status; CS, Clinical Stage; HL, Hodgkin’s lymphoma; NHL, Non-Hodgkin lymphoma; DLBCL, Diffuse large B-cell lymphoma; FL, Follicular lymphoma; HT, Height; BW, Body Weight; BSA, Body Surface Area; BMI, Body Mass Index

**Table2:** Comparison of peripheral blood and nutritional markers of each group

Factor	All (n=77)	Deficient (n=20)	Low or Normal (n=57)	P-value
White blood cell (×10^3^/μL)	6.0 (4.7–7.4)	6.0 (4.8–9.0)	6.0 (4.5–6.6)	0.48
Hemoglobin (g/dL)	12.1 (11.4–13.6)	11.6 (10.4–12.6)	12.5 (11.7–14.1)	0.017
Total lymphocyte counts (×10^3^/μL)	1,062 (704–1,760)	1,038 (438–1,306)	1,080 (756–1,798)	0.11
CD3^+^ lymphocyte (counts/μL)	761 (446–1,183)	711 (157–1,118)	762 (476–1,189)	0.27
CD4^+^ lymphocyte (counts/μL)	394 (255–616)	327 (107–657)	406 (273–600)	0.40
CD8^+^ lymphocyte (counts/μL)	284 (163–537)	232 (49–355)	287 (166–585)	0.088
CD20^+^ lymphocyte (counts/μL)	101 (42–168)	104 (18–172)	103 (49–161)	0.61
Albumin (g/dL)	4.0 (3.4–4.3)	3.4 (2.5–3.9)	4.1 (3.6–4.4)	0.0020
GPS (0/1/2)	39/19/19	5/4/11	34/15/8	0.0020
NLR	3.4 (2.3–5.4)	5.1 (2.7–11.4)	3.1 (2.3–4.5)	0.039
PLR	186.7 (114.5–299.3)	194.5 (108.8–476.7)	186.5 (121.2–257.1)	0.47
PNI	46.0 (34.7–50.4)	35.3 (27.1–46.5)	48.2 (40.4–52.0)	0.0020
CONUT	2.5 (1.0–5.7)	4.5 (2.0–8.0)	2.0 (1.0–4.0)	0.026

Variables that did not conform to a normal distribution are expressed as the median (interquartile range). GPS, Glasgow Prognostic Score; NLR, Neutrophile-lymphocyte ratio; PLR, Platelet-lymphocyte ratio; PNI, Prognostic nutrition index; CONUT, Controlling Nutritional Status

**Table3:** Linear regression analysis of serum Zn level and other factors

	Estimate	95%CI	S.D.	T-value	P-value
Age	0.0045	−0.24–0.24	0.12	0.037	0.97
Alb	9.51	3.48–15.53	3.02	3.14	0.0024
Hb	1.09	−1.11–3.30	1.10	0.98	0.32
NLR	-0.042	−0.25–0.17	0.10	–0.39	0.69

adjusted R-squared: 0.28, p-value: 0.00001 95% CI, 95% confidence interval; S.D., standard deviation; Alb, albumin; Hb, Hemoglobin level; NLR, Neutrophile-lymphocyte ratio
